# Current and future resources for functional metagenomics

**DOI:** 10.3389/fmicb.2015.01196

**Published:** 2015-10-29

**Authors:** Kathy N. Lam, Jiujun Cheng, Katja Engel, Josh D. Neufeld, Trevor C. Charles

**Affiliations:** Department of Biology, University of WaterlooWaterloo, ON, Canada

**Keywords:** functional metagenomics, metagenomic library, cosmid library, fosmid library, pCC1FOS, cloning bias, library bias, RK2

## Abstract

Functional metagenomics is a powerful experimental approach for studying gene function, starting from the extracted DNA of mixed microbial populations. A functional approach relies on the construction and screening of metagenomic libraries—physical libraries that contain DNA cloned from environmental metagenomes. The information obtained from functional metagenomics can help in future annotations of gene function and serve as a complement to sequence-based metagenomics. In this Perspective, we begin by summarizing the technical challenges of constructing metagenomic libraries and emphasize their value as resources. We then discuss libraries constructed using the popular cloning vector, pCC1FOS, and highlight the strengths and shortcomings of this system, alongside possible strategies to maximize existing pCC1FOS-based libraries by screening in diverse hosts. Finally, we discuss the known bias of libraries constructed from human gut and marine water samples, present results that suggest bias may also occur for soil libraries, and consider factors that bias metagenomic libraries in general. We anticipate that discussion of current resources and limitations will advance tools and technologies for functional metagenomics research.

## The challenges of constructing large-insert metagenomic libraries

Functional metagenomics involves isolating DNA from microbial communities to study the functions of encoded proteins. It involves cloning DNA fragments, expressing genes in a surrogate host, and screening for enzymatic activities. Using this function-based approach allows for discovery of novel enzymes whose functions would not be predicted based on DNA sequence alone. Information from function-based analyses can then be used to annotate genomes and metagenomes derived solely from sequence-based analyses. Thus, functional metagenomics complements sequence-based metagenomics, analogous to how molecular genetics of model organisms has provided knowledge of gene function that is widely applicable in genomics.

**Figure 1:**
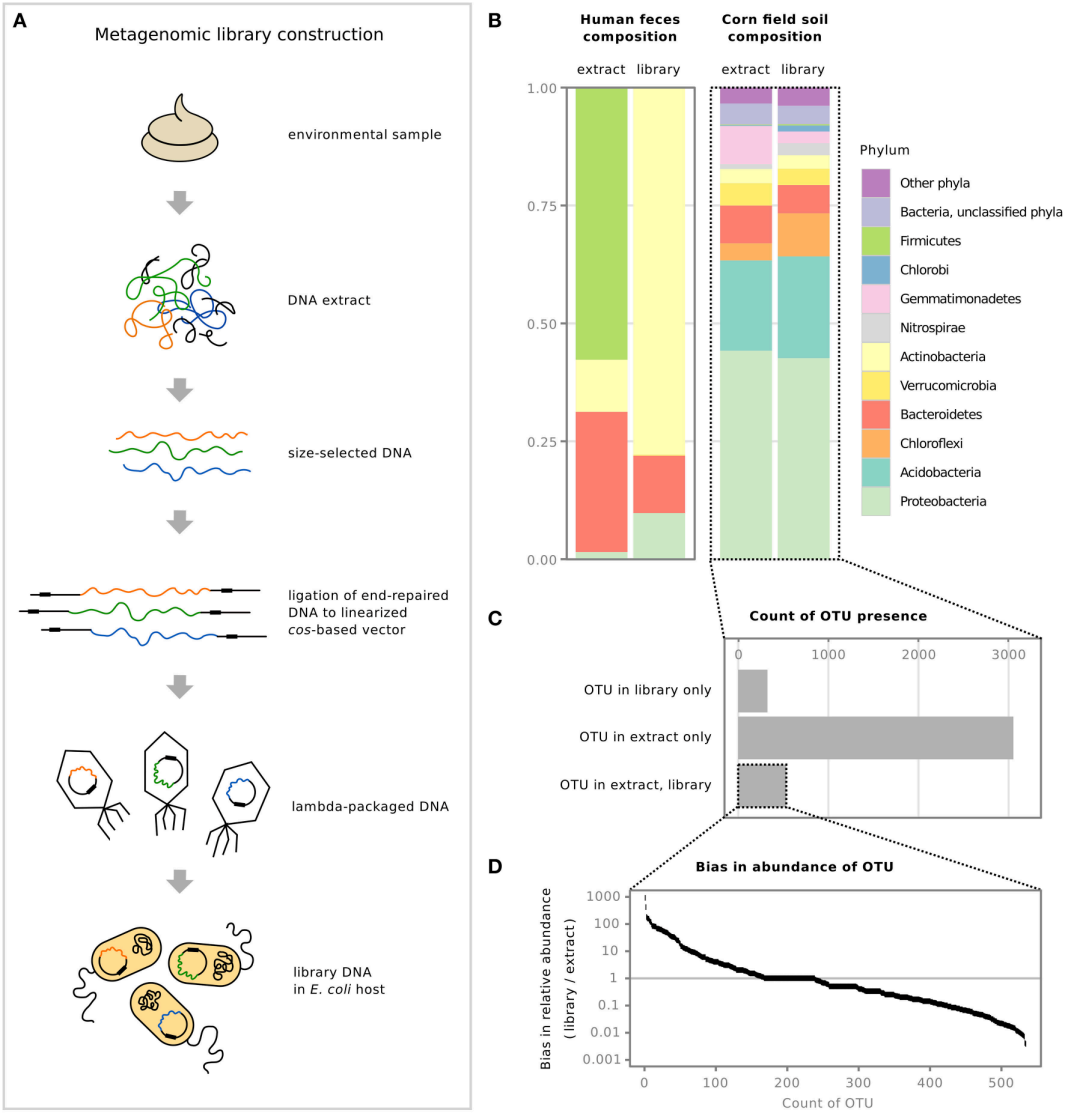
**Metagenomic libraries exhibit cloning bias when compared to the original environmental sample**. **(A)** Steps involved in the construction of a metagenomic library, from original environmental sample to the final library in the *E. coli* host (adapted from Lam and Charles, 2015). **(B)** Relative abundance of bacterial phyla from two previously constructed metagenomic libraries, a human fecal library (Lam and Charles, 2015), and a corn field soil library (Cheng et al., 2014), compared to their original sample DNA extracts. **(C)** Number of OTUs identified from corn field soil DNA extract and library, and whether the OTUs were present in the library sample only, the extract sample only, or present in both. **(D)** Examination of cloning bias by comparing the relative abundance of OTUs that were present in both the DNA extract and the cosmid library, shown on a log scale; horizontal line at 1 denotes equal relative abundance in both samples.

Functional metagenomics begins with the construction of a metagenomic library (Figure 1A). Cosmid- or fosmid-based libraries are often preferred due to their large and consistent insert size and high cloning efficiency. DNA is first extracted from the environmental sample of interest, then size-selected, end-repaired, and ligated to a *cos*-based vector, allowing packaging by lambda phage for subsequent transduction of *Escherichia coli* (Figure 1A). The resulting library contains relatively large insert DNA, typically 25–40 kb for *cos*-based vectors. With the steps involved, the construction of a metagenomic library can be laborious and time-consuming, requiring a high level of skill at the laboratory bench.

There are several technically challenging steps in library construction. First, the extracted DNA must be of sufficient length for efficient packaging into lambda phage heads (Parks and Graham, 1997). Extraction usually employs gentle lysis to avoid shearing DNA (Zhou et al., 1996) but even so it may be difficult to achieve large fragment sizes (Kakirde et al., 2010). We find that starting with crude DNA extracts containing at least ~75 kb fragments leads to high-quality libraries and it is crucial to check the fragment size range by pulsed-field electrophoresis before proceeding. A particularly useful and affordable molecular ladder for pulsed-field gels is self-ligated lambda DNA, which can be easily prepared and results in bands at approximately 50, 100, and 150 kb. A freeze-grinding step prior to extraction (Lee and Hallam, 2009) can substantially improve cell lysis. Although this step may fragment DNA (Brady, 2007), we find it does not hinder library construction, consistent with previous work showing that freeze-grinding results in minimal shearing (Zhou et al., 1996).

Extracts are often contaminated with compounds that co-purify with DNA, requiring additional purification steps that may lead to sample loss. Common contaminants in soil-derived DNA extracts are humic acids, which may interfere with enzymatic reactions (Tebbe and Vahjen, 1993). Non-linear electrophoresis is effective for contaminant removal (Pel et al., 2009) and generates purified and concentrated DNA suitable for PCR or metagenomic analysis (Engel et al., 2012), yet requires specialized equipment. We have found that for library construction, humic acids can simply be allowed to run off the gel during pulsed-field electrophoresis of crude extract for size-selection because they migrate much faster than large DNA fragments. Alternatively, to avoid contaminating the circulating buffer, electrophoresis can be paused after humic acids have formed a front, the part of the gel containing the humic acids excised, and then this region replaced with fresh gel (Cheng et al., 2014). Others have reported that contaminating nucleases are effectively inhibited by treating extracted DNA in an agarose plug with sodium chloride and formamide (Liles et al., 2008).

After the DNA has been size-selected and purified, it must be end-repaired and ligated to a desphosphorylated, blunt-ended vector. To ensure proper size range before ligation, the DNA can be checked for co-migration with the largest band of a lambda-HindIII ladder on an agarose gel (Brady, 2007) or the sample can be run on a pulsed-field gel for a more accurate size assessment. The end-repair is a challenging step because there is no simple way to confirm that ends are indeed blunt following the reaction. We use a small amount of the ligation to transform *E. coli* prior to the costly packaging step; resulting transformants indicate the presence of circular DNA molecules arising from ligation of successfully blunt-ended fragments. Though the ligation conditions may not favor formation of circular molecules, this is our best proxy for successful end-repair.

Other challenges include the sensitivity of packaging extracts and preparation of purified digested and dephosphorylated vector DNA for ligation. Although excellent commercial products are available for both, in-house vector preparation may still be required when specific expression hosts are to be used in functional screening outside the host range of available commercial vectors (Wexler et al., 2005; Craig et al., 2010; Troeschel et al., 2010; Cheng et al., 2014). The culminating step of library construction is the transduction of *E. coli*, and although it is possible to generate many thousands of clones with the first attempt, troubleshooting may be required to increase library size. When transduction results in a disappointingly small number of transductants (zero in the worst case!), it is not easy to determine the cause.

Indeed, metagenomic library construction is in many ways an art that takes time and practice to master. Given the substantial challenges and costs associated with library construction, as well as possible difficulties in obtaining rare environmental samples, a clear corollary is that we ought to find ways to maximize these valuable resources for shared benefit. In particular, collections of metagenomic libraries that can be used in a variety of hosts would be extremely valuable if able to be accessed by the scientific community. We have previously made our libraries publicly available (Neufeld et al., 2011) and we continue to advocate for increased sharing (Charles and Neufeld, 2015). Though there are obvious administrative obstacles, services such as Addgene (Herscovitch et al., 2012) may facilitate these efforts.

## Making the most of what we have: leveraging existing libraries

Due to the difficulties of library construction, commercial products that aid in generation of libraries are popular. Indeed, one widely used cloning-ready commercial vector is pCC1FOS (Genbank accession EU140751; Epicentre Biotechnologies). In recent years, as functional metagenomics has gained traction, metagenomic libraries from remarkably diverse environments have been constructed using pCC1FOS (Table 1). The pCC1FOS vector has several advantages. It carries a chloramphenicol resistance (*cat*) marker that is superior to the common ampicillin resistance (*bla*) marker, obviating the occurrence of satellite colonies associated with beta-lactamase secretion that can be problematic for the dense platings often required for library construction. In addition to an F plasmid *oriV* for single-copy maintenance, pCC1FOS also carries an *oriV* from the RK2 plasmid. The RK2 *oriV* is broad-host-range, conferring replication ability in diverse members of the *Proteobacteria* (Ayres et al., 1993), but requires the *trfA* gene product for replication and results in an estimated 15 copies per cell (Durland and Helinski, 1990). Though *trfA* is not carried by the fosmid, it can be provided in trans; notably, the commercial *E. coli* strain EPI300 (Epicentre Biotechnologies) carries *trfA* under the control of an inducible promoter that is advertised to increase copy number from 1 copy per cell to 10–200 copies. The strain likely possesses a *trfA* copy-up mutant allele under control of *araC-P*_*BAD*_, which is induced by L-arabinose (Wild et al., 2002). In the past, we preferred HB101 as a library host due to its receptiveness to transduction, but EPI300 appears to transduce at least as well as, if not better than, HB101. It also has the advantages of being an *endA1* mutant and supporting copy-number inducibility, allowing for less-degraded and higher-yield plasmid preparations.

**Table 1:** **Examples of metagenomic libraries constructed from diverse environmental samples using cloning vector pCC1FOS/pCC2FOS or derivatives**.

**Environment**	**Library vector; screening host, if relevant**	**References**
**HOST-ASSOCIATED ENVIRONMENTS**		
Bovine rumen	pCC1FOS; *E. coli*	Wang et al., 2013
Elephant feces	pCC1FOS; *E. coli*	Rabausch et al., 2013
Human distal ileum	pCC1FOS; *E. coli*	Cecchini et al., 2013
Human feces	pCC1FOS; *E. coli*	Jones et al., 2008
Human feces (pescatarian)	pCC1FOS; *E. coli*	Tasse et al., 2010
Marine sponge	pCC1FOS	Yung et al., 2009
Termite gut	pCC1FOS, pCC2FOS; *E. coli*	Warnecke et al., 2007; Liu et al., 2011
**EXTREME ENVIRONMENTS**		
Alaskan soil	pCC1FOS; *E. coli*	Allen et al., 2009
Alaskan floodplain soil	pCC1FOS; *E. coli*	Williamson et al., 2005
Antarctic Pennisula meltwater	pCC1FOS; *E. coli*	Ferrés et al., 2015
Glacial ice	pCC1FOS; *E. coli*	Simon et al., 2009
Hot spring sediment and biofilm	pCT3FK; *E. coli, Thermus thermophilus*	Leis et al., 2015
Hydrothermal fluids	pCC1FOS; *E. coli*	Böhnke and Perner, 2015
**MARINE OR FRESHWATER ENVIRONMENTS**		
Bog	pCC1FOS; *E. coli*	Sommer et al., 2010
Marine sediment	pRS44; *Pseudomonas fluorescens, Xanthomonas campestris*	Aakvik et al., 2009
Ocean tidal flat sediment	pCC1FOS; *E. coli*	Lee et al., 2006, 2015
Ocean water column	pCC1FOS	DeLong et al., 2006
River sediment	pCC1FOS; *E. coli*	Rabausch et al., 2013
**POLLUTED ENVIRONMENTS**		
Crude oil-contaminated shore	pMPO579; E. coli^*^	Terrón-González et al., 2013
Polluted river	pCC1FOS; *E. coli*	Vercammen et al., 2013
**AGRICULTURAL, ENGINEERED, OR OTHER ENVIRONMENTS**		
Activated sludge	pCC1FOS, pCC2FOS; *E. coli*	Suenaga et al., 2007; Zhang and Han, 2009
Compost, leaf branch	pCC1FOS; *E. coli*	Sulaiman et al., 2012
Compost, lumber waste	pCT3FK; *E. coli, Thermus thermophilus*	Leis et al., 2015
Compost, wood/plant debris/manure	pCC1FOS; *E. coli*	Ohlhoff et al., 2015
Decomposing leaf litter	pCC1FOS; *E. coli*	Nyyssönen et al., 2013
Orchard soil	pCC1FOS; *E. coli*	Donato et al., 2010
Sugarcane bagasse	pCC1FOS	Mhuantong et al., 2015

*Libraries that are based on the commercial pCC1FOS or pCC2FOS vector can be screened in any RK2-compatible host that expresses the trfA gene product required for the broad-host-range RK2 oriV origin of replication*.

* *modified strains derived from E. coli EPI300 to increase transcription*.

Despite its popularity, pCC1FOS has some disadvantages that make resulting libraries less versatile than they could be. First, pCC1FOS does not possess an *oriT* that would allow the fosmid to be efficiently transferred by conjugation, mediated by a helper plasmid, to other species or strains that may be more suitable for heterologous expression. To achieve conjugation capabilities, we have added the RK2 *oriT* to pCC1FOS (Lam and Charles, unpublished), as have others (Aakvik et al., 2009; Buck, 2012; Terrón-González et al., 2013). To enable conjugation after library construction has already taken place, others have retrofitted individual pCC1FOS-based clones with an *oriT* (Li et al., 2011; Buck, 2012). These modifications illustrate the need for fosmid and cosmid vector design to include the *oriT* so that duplication of work can be avoided. It is possible that transformation can be used to transfer libraries to other hosts, but only for recipients that are amenable to those techniques and that will not reject DNA that has been synthesized in *E. coli* due to the presence of host restriction-modification systems. In some cases, it will be desirable to modify these host strains by deleting the restriction-modification genes.

Given that the broad-host-range *oriV* is used to achieve a higher copy number in EPI300 expressing the *trfA* gene, another disadvantage of pCC1FOS is that *trfA* is not included on the vector. The consequence is that species that would otherwise be able to use the *oriV* cannot replicate pCC1FOS. It is not surprising then that for the vast majority of studies highlighted here (Table 1), *E. coli* was used as the screening host. This is a disadvantage for functional metagenomics as different clones can be isolated from the same metagenomic library when different screening hosts are used (Martinez et al., 2004; Craig et al., 2010). We found that using the legume-symbiont *Sinorhizobium meliloti* as a host results in a much greater diversity of clones than *E. coli* when screening our corn field soil metagenomic library for beta-galactosidase activity, though this greater diversity does not appear to be related to phylogenetic distance of the origin of the cloned DNA to the surrogate host (Cheng et al., in preparation). The importance of devising systems that allow for functional screening in diverse expression hosts has been reviewed by others (Uchiyama and Miyazaki, 2009; Taupp et al., 2011; Ekkers et al., 2012; Liebl et al., 2014), but what of the large number of libraries that have already been constructed? Can we make use of them for screening in non-*E. coli* hosts? The libraries listed in Table 1, as well as potentially many other metagenomic libraries constructed using pCC1FOS or derivatives, would be accessible to any RK2-compatible host if a copy of the *trfA* gene were also made available. This solution has already been applied: one group inserted the *trfA* gene into the chromosome of the *Gammaproteobacteria* species *Pseudomonas fluorescens* and *Xanthomonas campestris* for screening of libraries constructed using a pCC1FOS derivative (Aakvik et al., 2009). Another group inserted *araC-P*_*BAD*_-*trfA* into the *E. coli* EL350 chromosome to give copy number inducibility to the lambda Red recombineering strain (Westenberg et al., 2010). The introduction of *trfA* into RK2-compatible species is a straightforward way to expand the range of expression hosts for existing pCC1FOS-based libraries.

An alternative to inserting the *trfA* gene into desired expression hosts is to modify the vector for integration into the host genome, bypassing the requirement for *trfA*. This strategy has been employed to integrate clones into a target locus in the genome of the thermophile *Thermus thermophilus* for functional screening, by modifying pCC1FOS to include a selectable marker as well as regions for homologous recombination (Angelov et al., 2009). In our lab, pCC1FOS was modified to carry ΦC31 *att* sites (Heil and Charles, unpublished) for integrase-mediated site-specific recombination of cloned insert DNA into the genomes of landing pad strains, including *S. meliloti* and *Agrobacterium tumefaciens* (Heil et al., 2012). As a general strategy, however, chromosomal integration is potentially less useful than clone maintenance due to the difficulty in retrieving the integrated DNA for manipulation, including DNA sequence analysis, when non-arrayed (i.e., pooled) libraries have been screened.

## Knowing the extent of what we have: examining cloning bias

Beyond the practical questions of how to optimize vectors for library construction and how to maximize valuable existing libraries, there is a technical question that we find particularly interesting: how much of the sequence diversity present in original DNA extracts is captured in constructed libraries, and what affects this? Though not so much a concern for functional screens, it is interesting to consider the factors that influence library representativeness; elucidating these factors may lead to development of better strategies for accessing the full potential of environmental metagenomes. We previously used shotgun sequencing to examine bias in a human fecal library (Lam and Charles, 2015) and here we also present the results of 16S rRNA gene sequencing to examine bias in a corn field soil library (Cheng et al., 2014); see Supplementary Material for details. Both libraries were constructed using the RK2-based cosmid pJC8 (Genbank accession KC149513).

The bias discussed here is from comparing DNA extracted from the sample to the final cloned library DNA isolated from *E. coli* (Figure 1A). Analysis at the phylum-level showed that although the fecal library differed substantially in the relative abundance of phyla compared to its corresponding extract, the relative abundance of phyla in the corn field soil library seemed similar to its extract (Figure 1B). We present these results for the soil library but exercise caution in their interpretation as the majority of 16S rRNA gene sequences from the metagenomic library sample was *E. coli* contamination, despite treating the library cosmid DNA preparation with Plasmid-Safe DNase to remove host genomic DNA prior to PCR. After subtracting *E. coli* host sequences, approximately 30,000 sequences remained to represent the metagenomic library (see Supplementary Material for details). The high level of host contamination could be due to preferential amplification of template during PCR based on differences in DNA conformation: though present in very small quantities, linear DNA may be more efficiently amplified over supercoiled or closed circular plasmid DNA (Chen et al., 2007). This issue of *E. coli* host contamination in 16S rRNA gene analysis needs to be addressed for future examination of bias in metagenomic libraries.

When we examined the soil samples more closely, we found that the similarity of the library and extract at the phylum level does not extend to the “species” level: examination of the individual OTUs in each sample revealed that only a small fraction of OTUs were shared between the library and original sample (Figure 1C). Interestingly, our analysis indicated that there were a number of OTUs in the library that were not identified in the extract sample (Figure 1C) and although this number is halved when the library data are compared to extract data that have not been rarefied (data not shown), they nevertheless remain, indicating that these OTUs are either extremely rare in the original sample and their DNA is preferentially cloned or that the identification of these OTUs is due to sequencing errors. A further analysis of the OTU fraction that is shared between extract and library samples shows a large range in the bias in relative abundance of each OTU, with some OTUs exhibiting ~1000-fold overrepresentation and others ~1000-fold underrepresentation in the library (Figure 1D). While there may be concern that 16S rRNA gene profiles of libraries compared to extracts may not provide an accurate comparison of cloned DNA content in general, we have previously shown from analysis of shotgun sequence data that for large-insert RK2 *oriV*-based cosmid libraries, 16 S rRNA gene content tracks well with genomic content (Lam and Charles, 2015). The analysis of the corn field DNA extract and corresponding metagenomic library suggests that though the overall relative abundance of phyla may remain similar, bias is occurring on the level of individual OTUs.

The fact that certain taxa are under- or overrepresented might not pose a barrier to screening, but it may be useful to know what sequences are not likely to be captured in libraries. Several studies that have compared shotgun sequencing of original samples to corresponding metagenomic libraries from marine water (Temperton et al., 2009; Ghai et al., 2010; Danhorn et al., 2012), as well as our own comparative work on feces (Lam and Charles, 2015), have shown that AT-rich sequences are underrepresented in libraries. Our analysis—in which we compared promoter consensus sequences between extract and library samples—lends support to the hypothesis that the bias is related to spurious transcription of metagenomic DNA from AT-rich sequences recognized as σ^70^ promoters in the *E. coli* library host (Lam and Charles, 2015) although other factors may be contributing, such as gene product toxicity (Sorek et al., 2007). Notably, we have shown that DNA fragmentation is not a cause of bias (Lam and Charles, 2015). The specific factors affecting the “clonability” of DNA, and the mechanisms that lead to DNA exclusion, still need to be experimentally determined.

The stability of foreign DNA in *E. coli* is influenced by the vector copy number and, as a result, single-copy fosmids may be ideal as the library backbone (Kim et al., 1992), although the success of some functional screens may be dependent on a higher gene dose. Plasmid vectors that are not *cos*-based provide an alternative where cloning is substantially less difficult as large-fragment DNA need not be isolated and packaging and transduction are not required; the disadvantages, however, are that a smaller insert size means that larger operons will not be intact, and if the plasmid has a high copy number—true of conventional cloning vectors—this may lead to greater insert instability and exclusion (Lam and Charles, 2015). Other alternatives to fosmid vectors include BACs (Kakirde et al., 2011), which have the ability to capture even larger insert sizes at approximately 100 kb on average (Kakirde et al., 2010), and linear vectors, which may provide exceptional stability (Godiska et al., 2010). However, *cos*-based vectors are likely to remain popular for their advantages: the availability of high-quality commercial packaging extracts, greater efficiency of transduction over transformation, and decreased probability of insert concatemers due to the phage head upper size limit. Though there exists variety in library cloning vectors, further work is required to understand how and to what extent cloning vector choice and strategy impacts library sequence bias.

## Concluding remarks

Depending on the target activity, functional screens can exhibit a low hit rate (Uchiyama and Miyazaki, 2009) the reasons for which might include barriers at the level of both transcription and translation. Improving *E. coli* as a screening host to address these problems will likely improve future hit rates. Examples include introducing heterologous sigma factors to guide RNA polymerase to otherwise untranscribed regions (Gaida et al., 2015), employing T7 RNA polymerase to help drive transcription (Terrón-González et al., 2013), as well as forming hybrid ribosomes (Kitahara et al., 2012) that may influence expression. Nevertheless, it will be important to move beyond *E. coli* into different screening hosts, particularly for the complementation of mutant phenotypes not possible in *E. coli*. The identification of obstacles to cloning and screening will aid in the development of new tools and technologies for functional metagenomics (Engel et al., 2013), providing us with greater reach in terms of what we are able to gather from functional screens. The refinement of methods will be crucial in bioprospecting for novel enzymes and compounds as well as for the determination of gene function that will guide the development of reliable models of microbial ecosystem functioning.

## Author contributions

KL and TC conceived the ideas. JC prepared DNA from the soil-related samples. KE carried out V3 region PCR on the soil-related samples and managed sequencing sample submission. KL analyzed the sequence data, made the figures, performed the literature review, and wrote the paper. TC, JN, JC, and KE revised the manuscript. TC and JN provided reagents and materials. All authors read and approved the manuscript.

## Funding

Research funding was provided by a Strategic Projects Grant (381646–09) from the Natural Sciences and Engineering Research Council of Canada, by Genome Canada for the project “Microbial Genomics for Biofuels and Co-Products from Biorefining Processes,” and by a University of Waterloo CIHR Research Incentive Fund. KL was supported by a CGS-D scholarship from the Canadian Institutes of Health Research.

### Conflict of interest statement

The authors declare that the research was conducted in the absence of any commercial or financial relationships that could be construed as a potential conflict of interest.
