# Supplementary material for: Current and future resources for functional metagenomics
Source: Front Microbiol. 2015 Oct 29;6:1196. doi: 10.3389/fmicb.2015.01196 (PMC4625089; doi:10.3389/fmicb.2015.01196)
Supplement: Supplementary file 1 [file DataSheet1.DOCX]

**Supplementary Material**

**Current and future resources for functional metagenomics**

Kathy N. Lam^1^, Jiujun Cheng^1^, Katja Engel^1^, Josh D. Neufeld^1^, and Trevor C. Charles^1^*

^1^Department of Biology, University of Waterloo, Waterloo, ON, Canada

***Correspondence:**

Trevor C. Charles, Department of Biology, University of Waterloo, Waterloo, ON, Canada tcharles@uwaterloo.ca

**Supplementary methods**

***Data availability.*** Sequence data for the CLGM1 human gut DNA extract and corresponding metagenomic library (NCBI BioSample SAMN02324081) have previously been made available (Lam and Charles, 2015). Sequence data for the 12AC corn field soil DNA extract and corresponding metagenomic library (NCBI BioSample SAMN02324088) previously constructed (Cheng et al., 2014) have been deposited at NCBI SRA; accession numbers are SRX1015944 and SRX1015946 for the extract and cosmid library, respectively. In addition, sequence data and other relevant data may be accessed at <http://www.cm2bl.org/~data>

***16S rRNA analysis for 12AC soil DNA extract and metagenomic library.*** Construction of the 12AC library was previously described (Cheng et al., 2014). Crude DNA extract of corn field soil was purified using the SCODA method (Engel et al., 2012). Cosmid library DNA was miniprepped from *E. coli* HB101 using a GeneJet Plasmid Miniprep kit (Thermo Scientific). Cosmid DNA was treated with Plasmid-Safe ATP-dependent DNase according to the supplier's recommendations (Epicentre Biotechnologies). PCR was carried out on the samples as previously described, using bacterial V3-specific primers 5'CCTACGGGAGGCAGCAG and 5'ATTACCGCGGCTGCTGG (Bartram et al., 2011). Amplicons were sequenced at the NRC-PBI Saskatoon Research Facility (Saskatoon, Canada) using the Illumina GAIIx platform. Paired-end sequences were assembled using PANDAseq version 2.8 (Masella et al., 2012) using default parameters; 1,823,112 and 1,886,370 sequences were assembled for the extract and cosmid library sample, from an input of 1,960,793 and 2,035,138 paired-end sequences, respectively. *E. coli* sequences were filtered out, using a criterion of 100% identity to *E. coli* MG1655 (the closest sequenced relative of HB101), resulting in 233 sequences removed from the extract sample and 1,453,806 sequences removed from the cosmid library sample. Sequences were subsequently processed via AXIOME2 (Lynch et al., 2013) running QIIME version 1.9, specifying UPARSE (USEARCH version 7.0) to cluster the sequences using default parameters and the RDP classifier version 2.2 trained with the Greengenes database version 13.8 to classify defined OTUs. From the resulting OTU table, *E. coli* was filtered a second time by manually removing OTUs classified as *Enterobacteriaceae*, which consisted of 109 sequences from the extract sample and 335,994 sequences from cosmid library sample. The extract sample was then rarefied using QIIME to match the cosmid library, retaining ~30,000 sequences for each sample, altogether comprising ~4000 OTUs.
